# MRI of Arterial Flow Reserve in Patients with Intermittent Claudication: Feasibility and Initial Experience

**DOI:** 10.1371/journal.pone.0031514

**Published:** 2012-03-08

**Authors:** Bas Versluis, Marjolein H. G. Dremmen, Patty J. Nelemans, Joachim E. Wildberger, Geert-Willem Schurink, Tim Leiner, Walter H. Backes

**Affiliations:** 1 Department of Radiology, Maastricht University Medical Center, Maastricht, The Netherlands; 2 Department of Surgery, Maastricht University Medical Center, Maastricht, The Netherlands; 3 Department of Epidemiology, Maastricht University Medical Center, Maastricht, The Netherlands; 4 Cardiovascular Research Institute Maastricht (CARIM), Maastricht, The Netherlands; 5 Department of Radiology, University Medical Center Utrecht, Utrecht, The Netherlands; University of Las Palmas de Gran Canaria, Spain

## Abstract

**Objectives:**

The aim of this work was to develop a MRI method to determine arterial flow reserve in patients with intermittent claudication and to investigate whether this method can discriminate between patients and healthy control subjects.

**Methods:**

Ten consecutive patients with intermittent claudication and 10 healthy control subjects were included. All subjects underwent vector cardiography triggered quantitative 2D cine MR phase-contrast imaging to obtain flow waveforms of the popliteal artery at rest and during reactive hyperemia. Resting flow, maximum hyperemic flow and absolute flow reserve were determined and compared between the two groups by two independent MRI readers. Also, interreader reproducibility of flow measures was reported.

**Results:**

Resting flow was lower in patients compared to controls (4.9±1.6 and 11.1±3.2 mL/s in patients and controls, respectively (p<0.01)). Maximum hyperemic flow was 7.3±2.9 and 16.4±3.2 mL/s (p<0.01) and the absolute flow reserve was 2.4±1.6 and 5.3±1.3 mL/s (p<0.01), respectively in patients and controls. The interreader coefficient of variation was below 10% for all measures in both patients and controls.

**Conclusions:**

Quantitative 2D MR cine phase-contrast imaging is a promising method to determine flow reserve measures in patients with peripheral arterial disease and can be helpful to discriminate patients with intermittent claudication from healthy controls.

## Introduction

The hemodynamic significance of obstructive arterial lesions in peripheral arterial disease (PAD) can be assessed by blood flow velocity measurements [1], [2], [3], [4], [5]. Nevertheless, in patients with intermittent claudication - stage II PAD according to the Fontaine classification - blood flow at rest might be near-normal depending on the severity of the obstructive lesion, or recover quickly to near-normal values after exercise due to collateral artery formation [6], [7], [8], [9], [10], [11], [12], [13]. These (near-)normal resting flow values explain the absence of clinical symptoms at rest in intermittent claudication. Symptoms in these patients, however, characteristically appear during physical exercise when the flow demands towards the lower extremity increase. Therefore the flow reserve, either defined as the absolute difference or relative value between maximum hyperemic flow and autoregulated resting flow, is expected to be compromised in patients with intermittent claudication [10], [14], [15]. Arterial flow reserve correlates well with subjective severity of symptoms in intermittent claudication and is less hampered by day-to-day variations than resting flow [14]. Also, flow reserve stays compromised for a prolonged period of time during the process of collateral formation [9]. Moreover, it does not depend on the physical capabilities of the patient, such as with physical exercise tests and ankle-brachial index measurements. Flow reserve might therefore be a valuable additional objective measure of disease severity in PAD in those patients with normal resting flow values, in order to discriminate between hemodynamically significant and insignificant obstructive arterial lesions. Flow reserve could also be useful for therapy monitoring, since physical exercise tolerance is closely correlated with the flow reserve, in contrast to resting flow levels [7], [8], [9].

Flow reserve is usually measured with duplex ultrasonography. This method, however, is limited for the peripheral arteries by a relatively large interobserver variability during reactive hyperemic conditions, which are needed to determine the flow reserve [16], [17]. Other techniques to measure blood flow are Laser Doppler flowmetry, contrast-enhanced ultrasound (CEUS) and intra-arterial catheter based flow measurements [1], [9], [10], [14], [18]. Unfortunately, these methods either lack the ability to directly measure flow in large, non-superficial, conduit arteries, are prone to poor interobserver variability, or are not suitable for repeated measurements, as in the context of therapy monitoring [9], [14], [16]. A promising alternative method to measure flow in the peripheral arteries, is MRI-based quantitative cine phase-contrast imaging (PCI) [2], [3], [4], [19], [20]. Cine PCI can easily be combined with standard contrast-enhanced MR angiograpy (CE-MRA) of the peripheral vasculature and therefore has the major advantage to provide highly accurate morphologic information [21], [22] together with relevant functional information in a single examination.

The purpose of the current study was (i) to develop a MRI method for the determination of vascular flow reserve in the popliteal artery (PA), using quantitative velocity encoded 2D MR cine PCI flow waveform measurements at rest and during reactive hyperemia, (ii) to evaluate the potential value of this method to discriminate patients with intermittent claudication from healthy control subjects, and (iii) to determine the reproducibility of measuring resting flow and flow reserve with cine PCI.

## Materials and Methods

### Subjects and Ethics statement

Ten consecutive non-diabetic patients (mean age ± SD: 66.5±9.9 years; 8 males and 2 females) with clinical symptoms of PAD (Fontaine stage II; intermittent claudication with no signs of critical ischemia [23]) with suspected arterial lesions of the superficial femoral artery (SFA) by duplex ultrasonography (peak systolic velocity (PSV) ratio >2.0 [24]) were included, as well as 10 healthy control subjects with no known PAD symptoms (age 24.1±2.2 years; 3 males and 7 females). Exclusion criteria were hemodynamic instability, contraindications for MRI (i.e. claustrophobia, known gadolinium based contrast agent allergy, or low estimated glomular filtration rate (<30 mL/kg/1.73 m^2^)). The institutional medical ethics committee (METC azM/UM) approved the study and all subjects gave written informed consent before inclusion.

### MRI protocol

All subjects underwent cine PCI measurements in the PA to obtain flow waveforms. Cine PCI was combined with CE-MRA of the peripheral arteries as part of the clinical routine examination. A schematic overview of the scan protocol is given in figure S1. All examinations were performed on a 1.5-T MRI system (Intera, Philips Medical Systems, Best, The Netherlands). For signal reception we used a dedicated 12-element phased-array peripheral vascular coil with a craniocaudal coverage of 128 cm (Philips Medical Systems, Best, The Netherlands). Subjects were imaged in the supine position. All subjects were in this position for approximately 30 minutes before the first flow measurement was started. During this time a three-station CE-MRA was acquired in patients, using a fixed dose of 10 mL gadofosveset trisodium (Ablavar®, Lantheus Medical Imaging, Billerica, MA) as contrast agent.

In patients, flow was measured in the PA of the most symptomatic lower extremity. In all healthy controls flow was measured in the right PA. Postischemic reactive hyperemia in the lower leg was provoked using an inflatable cuff, ensuring total arterial occlusion (Medrad, indianola, PA). The cuff was placed at mid-thigh level and manually inflated to suprasystolic values (>50 mmHg above brachial systolic blood pressure) during 6 minutes. During cuff inflation, flow in the PA was measured with cine PCI to check that total arterial occlusion of the upper leg was achieved. PA was chosen as the vessel of interest as the PA is the most distal artery with sufficiently large caliber to measure flow by PCI with a spatial resolution that enables fast cine PCI measurements [19], [20].

#### Survey

A non-enhanced time-of-flight (TOF) scan of the pelvic, upper and lower leg station was acquired to prescribe the imaging volumes of interest for morphological and functional imaging. A turbo field echo (TFE) pulse sequence was used with a 180° inversion prepulse to suppress stationary tissue. Thirty-one axial slices per station were acquired with 3.3-mm slice thickness and 11-mm interslice gap, and an inferiorly concatenated saturation band. The standard quadrature body coil was used for signal transmission and reception. For the positioning of the 3D CE-MRA volumes maximum intensity projections (MIPs) were generated in 3 orthogonal directions.

#### CE-MRA

A three-station 3D FFE CE-MRA sequence was performed as previously described [21], [25]. Acquisition parameters were as follows: TR 4.8 ms, TE 1.45 ms, flip angle 40°, FOV 470 mm, matrix 480, and voxel dimensions (reconstructed) 0.92×0.92×1.20 mm. Prior to contrast medium administration, a non-enhanced ‘mask’ image data set was acquired with exactly the same acquisition parameters as the CE-MRA, enabling background tissue suppression by image subtraction. For cine PCI we used a 2D FFE scan technique with the following acquisition parameters: TR 9.7 ms, TE 5.8 ms, flip angle 30°, FOV 380 mm, matrix 384, and voxel dimensions (reconstructed) 0.99×0.99×6.00 mm [26], [27], [28]. Fifteen dynamic phases were acquired during the cardiac cycle. To focus on the peak velocity and flow, the phase encoding velocity (VENC) was set to 100 cm/s in the craniocaudal direction, as published before [2], [29]. Vector cardiography (VCG) triggering was used for retrospective cardiac synchronization. Parallel imaging (sensitivity encoding, SENSE) was applied to reduce scan time (SENSE factor 2 in the anterior-posterior direction). At a mean heart rate of 60 beats per minute, the nominal acquisition time was 1 minute per dynamic acquisition. Coronal and sagittal MIP reconstructions of CE-MRA images were used for accurate positioning and angulation of the slice, perpendicular to the direction of the PA (see figure S2), for cine PCI measurement.

### Flow analysis

Two independent MRI readers, blinded for each other's results, analyzed all datasets. Modulus and phase images were reconstructed from the cine PCI data. MRIcro (MRIcro, http://www.mricro.com/) was used as the image software application to manually draw a region of interest (ROI) covering the entire visible cross-section of the PA on each of the 15 reconstructed modulus images spanning the cardiac cycle. These regions of interest (ROIs) were analyzed, using self-written software code (Matlab, The Mathworks inc., Natick, MA) to obtain flow waveforms from the phase images [27]. Phase images and flow waveforms were visually analyzed to detect possible aliasing due to a low phase encoding velocity. Flow waveforms were used to determine flow in mL/s by integrating the 2D velocity profile over the cross-section of the artery. For this study, we primarily focused on the vascular stress condition, and therefore the arterial peak flow (related to peak velocity) was the primary functional quantity measured, rather than mean flow or velocity, cross-sectional luminal area or other velocity measures, as peak flow is physiologically the most relevant quantity and known to be most reproducible [2]. Mean flow, peak velocity and peak area were nevertheless determined and can be found in appendix S1.

Resting flow was defined as the average flow of three consecutive acquisitions at rest prior to cuff inflation. Absolute flow reserve was defined as the absolute difference between maximum hyperemic flow after cuff release and resting flow, whereas the relative flow reserve was defined as the ratio between these two measures. Also the time-to-peak (TTP), defined as the time needed to reach maximum hyperemic flow after cuff release, and time-to-recovery (TTR), defined as the time needed for flow to recover towards resting values after cuff release, were determined. An overview of the different flow parameters is illustrated in figure S3. Flow values were recorded as the average flow over a single dynamic measurement (approximately one minute at a heart rate of 60 beats per minute). For example, if the maximum hyperemic flow was observed during the first measurement after cuff release, a TTP of 30 seconds was recorded. If the maximum hyperemic flow was observed during the second measurement, a TTP of 60+30 = 90 seconds was recorded. TTR was determined similarly.

### Statistical analysis

Statistical analysis was performed with commercially available statistical software (SPSS 16.0, SPSS Inc., Chicago, IL). For the small groups a non-parametric 2-samples test (Mann-Whitney test) was applied to compare measures between patients and healthy controls. P<0.05 was considered statistically significant. Data of both MRI readers were averaged for the comparison between patients and healthy controls.

The variation of consecutive flow measurements at rest and the interreader reproducibility of resting flow and flow reserve measures were calculated. To determine interreader reproducibility, two measures of agreement were determined, being the coefficient of variation (CV in %) and the repeatability coefficient (RC). In addition, the intraclass correlation coefficient (ICC) was determined as a parameter of reliability. The CV represents the relative variation within subjects and was derived by dividing the overall within-subject standard deviation (SD_ws_) by the mean measurement value over all subjects. The RC gives the smallest noticeable difference that can be detected beyond measurement error and is defined as 1.96⋅√2⋅SD_ws_ [30], [31]. In other words, the difference between 2 measurements in the same subject is expected to be less than the RC in 95% of the observations in cases where the measured quantity remains unchanged over time. A value of RC lower than the absolute difference between mean values in patients and healthy controls indicates good agreement. The ICC, the fraction of total variance due to variation between subjects, rather than measurement error, was calculated using a 1-way random model, according to ICC = SD_bs_^2^/(SD_bs_^2^+SD_ws_^2^), where SD_bs_ represents the standard deviation between subjects. If the measurement error is small compared to the variation between subjects, the ICC approaches 1, i.e. reliability is very high. Interreader reproducibility was determined for the resting flow and both flow reserve measures.

## Results

### Subjects

All subjects included underwent cine PCI to obtain flow waveforms as planned and without experiencing side effects or adverse events. Total arterial occlusion during cuff compression could be confirmed by cine PCI measurements in all subjects. All cine PCI images were of sufficient quality for quantitative analysis and no aliasing was detected. The average heart rate (mean ± SD) in patients was 67±9 beats per minute (bpm), versus 62±11 bpm in healthy controls (no significant difference, p = 0.23).

### CE-MRA

CE-MRA revealed significant stenosis (>50%) of the SFA in 6 out of 10 patients, whereas long occlusions of the SFA were found in the remaining patients. Large collateral arteries were found in those patients with SFA occlusions, whereas in patients with significant stenosis of the SFA there were no prominent collateral arteries. Iliac artery vessel wall irregularities were found in 3 out of 10 patients. The remaining patients had no signs of obstructive lesions of the iliac arteries. At least 2 out of 3 main conduit arteries of the lower leg were free from obstructive lesions in all patients. CE-MRA revealed no signs of PAD in any of the control subjects.

### Patients versus healthy controls

Representative phase-contrast images (modulus and phase), cross sectional velocity 2D velocity profiles and flow waveforms at rest and during reactive hyperemia in a patient and healthy control subject are shown in figure S4 and S5 respectively. Flow increased during reactive hyperemia in both patients and healthy controls. Flow waveforms under resting conditions were either tri- (n = 2), bi- (n = 2) or monophasic (n = 6) in patients and triphasic in all healthy controls. During reactive hyperemia flow waveforms became monophasic for all patients, as seen in figure S5, while flow waveforms remained triphasic for all healthy controls. Figure S6 shows temporal variations in flow before and after provoking reactive hyperemia in a patient and a healthy control.

Absolute flow values at rest and during reactive hyperemia are given in table S1. On average, resting flow in patients was less than half of the values measured in healthy controls (p<0.01). Maximum hyperemic flow and absolute flow reserve in patients were also less than half the values found in healthy controls (p<0.01). For relative flow reserve, on the other hand, there were no significant differences between patients and healthy controls (p = 0.81).

Figure S7 shows the distribution of resting flow and absolute flow reserve values in patients. The upper and lower limits represent mean values ±2·SD in healthy controls. Resting flow was lower than the lower limit of healthy controls (i.e. mean – 2·SD) in 4 patients. Absolute flow reserve revealed lower values than the lower limit in 6 patients, of whom 2 patients had normal resting flow values (see cross-table of figure S7). In 4 patients both resting flow and flow reserve values were within the upper and lower limits as encountered in control subjects.

TTP was 65±52 (mean ± SD) seconds in patients and 35±29 seconds in healthy controls. TTR was 360±212 and 254±96 seconds in patients and healthy controls, respectively. These results for TTP and TTR indicate a trend towards slower responses in patients (p = 0.15 and p = 0.16 for TTP and TTR, respectively).

### Vascular lesions and flow measures

Resting flow in patients with an occlusion of the SFA was significantly lower compared to those with a significant stenosis (p = 0.02). Three out of 4 patients with an occlusion of the SFA had resting flow values lower than the lower limit of healthy controls.

Maximum hyperemic flow, absolute and relative flow reserve measures did not differ significantly between patients with occlusions and significant stenosis (p = 0.15, p = 0.72 and p = 0.63 respectively). Absolute flow reserve was lower than the lower limit of healthy controls in 3 out of 4 patients with a SFA occlusion and 3 out of 6 patients with a significant stenosis of the SFA.

### Reproducibility

The variation between the three consecutive resting flow measurements was 4.2% in patients and 6.6% in healthy controls. Interreader reproducibility values of the flow measures are given in table S1. Interreader reproducibility for resting flow and flow reserve measures was high in both patients and healthy controls, with a CV below 10% for all flow measures in both groups (table S1). The RC values were smaller than the difference between PAD patients and healthy controls for the resting flow, maximum hyperemic flow, and the absolute flow reserve measures, but not for relative flow reserve.

## Discussion

In this study we describe a simple MRI-based cine phase contrast imaging method that can be used to determine flow reserve measures in the popliteal artery in a vascular stress condition. Our initial results indicate that this a stable method that seems to be more sensitive in detecting peripheral arterial disease than resting flow measurements only.

Objective assessment of the functional consequences of stenoses and occlusions in the peripheral arterial tree remains an area of high interest for physicians dealing with PAD patients. Currently, the ankle-brachial index (ABI) measurement is the most recognized and most widely applied functional measurement used for diagnosis and therapy monitoring [5], [32], [33]. ABI measurements, however, are hampered by poor reproducibility [14], [34]. Besides, in patients with heavily calcified arteries in the lower leg, as frequently seen in patients with PAD, the ABI cannot be determined accurately or not be determined at all [14], [34], [35].

The current study demonstrates that it is feasible to combine the ability of MRI to depict peripheral vascular anatomy with functional information (i.e. hemodynamic consequences) of popliteal artery flow reserve. Combining these two measurements in one examination is of high clinical interest, as it would potentially enable more objective assessment of PAD in patients suspected of having PAD, but also in the context of evaluating novel therapeutic strategies for PAD.

### Patients versus healthy controls

Maximum hyperemic flow and absolute peak flow reserve were approximately 50% and significantly lower in patients compared to healthy controls. This shows that flow reserve measures can be used to determine the hemodynamic consequences of obstructive arterial lesions in the superficial femoral artery in PAD patients. These results are in line with previous studies and prove that the concept of blood flow reserve, originally introduced by Coffman and Gregg in 1960 for the coronary arteries [15], also seem to apply to peripheral arteries [9], [10], [14], [36], [37]. Lower flow reserve in the popliteal artery of PAD patients can be explained by the presence of obstructive arterial lesions, resulting in a decrease of the pressure gradient distal to the vascular lesion and thereby a decrease of the maximum hyperemic flow distal to these lesions [37].

An important observation is that, at group level, resting flow in this study was significantly reduced in patients with intermittent claudication, which concurs with previous studies [2], [29]. This might suggest that resting flow alone already provides sufficient functional information on the hemodynamic significance of obstructive arterial lesions in intermittent claudication, without the need for flow reserve assessment. However, when looking at the resting flow values of individual patients (figure S7), it becomes clear that only four patients (40%) had resting flow values below the mean flow −2·SD in healthy controls. Three out of these four patients had a long occlusion of the SFA at CE-MRA. Regardless of the small number of patients, these initial results show that the type of vascular obstruction (i.e. stenosis or occlusion) affects resting flow. Although resting flow of these patients was lower than the lower limit in healthy controls, flow was still sufficient to sustain minimal perfusion of the lower leg as there were no clinical signs of ischemia at rest. Absolute flow reserve, on the other hand, was below the mean value in healthy controls – 2·SD in six (60%) patients and was therefore able to identify two more patients with PAD compared to resting flow alone. Of these six patients, three suffered from an occlusion of the SFA and three had a significant stenosis of the SFA on CE-MRA. In the four remaining patients both resting flow and flow reserve appeared normal. This suggests that either no hemodynamically significant obstructive arterial lesions were present, or flow through the collateral arteries mitigated the effects of the stenoses.

Our initial results also indicate that for patients with (near-)normal resting flow values, assessment of the flow reserve might provide important additional functional information with respect to the hemodynamic significance of the arterial lesions, allowing to discriminate patients with intermittent claudication from healthy controls . Further studies with larger populations will be needed to determine the additional clinical value of flow reserve in the assessment of the hemodynamic significance of obstructive arterial lesions, both in patients with intermittent claudication and more severe stages of PAD.

Relative flow reserve, also known as the dimensionless flow reserve ratio in literature [37], was not significantly different between patients and healthy controls in this study, although we expected it to be impaired in patients. This discrepancy can be explained by the fact that resting and maximum hyperemic flow in patients as well as volunteers were reduced to approximately the same extent, resulting in a ratio for patients comparable to that in healthy controls. Based on our results there seems no clinical value of the relative flow reserve.

TTP and TTR did not significantly differ between patients and volunteers. However, when looking at the absolute values, it becomes clear that TTP and TTR are prolonged in patients, as we expected. However, the fact that we did not find significant differences in TTP and TTR between patients and healthy controls might be due to the relative small number of subjects. In addition, the low temporal resolution of approximately one minute per acquisition probably had a negative influence upon the discriminative ability for TTP and TTR as well. Previous studies on microvascular reactions during reactive hyperemia found prolonged peak and recovery times for microvascular flow between PAD patients and controls and the same phenomenon might be expected for macrovascular flow [38], [39], [40].

### Reproducibility

Flow at rest was measured three times to determine an average resting flow. The variation between consecutive flow measurements at rest was very low in both patients (4.2%) and healthy controls (6.6%). This means that resting flow in a lying subject is relatively stable over time.

Resting flow and flow reserve measures (maximum hyperemic flow, absolute and relative flow reserve) all showed a high interreader reproducibility, with a interreader CV<5% for most measures (with exception of the absolute flow reserve in patients with a CV<10%). This indicates a low observer dependency of the measurements, which is desirable for clinical application in therapy monitoring and follow-up studies. The interreader CV is comparable to the variation of the (resting) flow measurements.

### Peak versus mean flow

In this study peak flow values were preferred over mean flow values. Peak flow, the maximum flow during systole, is an attractive flow measure, as it correlates with the ABI and is an important determinant of the systolic blood pressure [41]. The decision to use peak flow was based on previous results, suggesting that peak values are more reproducible in cine PCI of the popliteal artery and show larger differences between PAD patients and healthy controls [2]. Results on mean flow, peak velocity and peak area are presented in table S2 and discussed in appendix S1.

It would be interesting to see how well MRI derived flow reserve correlate and reproducibility measures compare with duplex ultrasonography derived flow measurements. Such a comparison, however, was beyond the scope of the current feasibility study and requires a larger sample size and patient population. Besides, at present flow reserve is not yet routinely used in clinical practice for establishing the diagnosis and severity of PAD, neither using duplex ultrasonography nor MRI.

### Spatiotemporal resolution

Previously published cine PCI protocols that measure flow reported a temporal resolution of 2 minutes or more [2], [42], [43], [44]. We believe that this temporal resolution would have been insufficient to accurately measure the maximum flow during reactive hyperemia, as it is known that the maximum flow after cuff release is reached within one minute [45]. Therefore, the temporal resolution of cine PCI measurements in the current study was improved by using parallel imaging (sensitivity encoding, SENSE [46], [47]). Prakash et al [47] found good agreement between conventional cine PCI measurements and flow measurements with a parallel imaging acceleration factor of 2, resulting in approximately 50% reduction in scan time. The results of our study show that in healthy controls the maximum hyperemic flow indeed was reached within one minute for every subject. To accurately measure the time-to-peak value in healthy subjects, and probably patients with less severe forms of PAD, further improvement of the temporal resolution is required. In PAD patients however, the time-to-peak was markedly longer, and in most patients the maximum hyperemic flow was not reached during the first measurement after cuff deflation. The currently used temporal resolution therefore seems to be sufficient for the application of this method in patients. Nevertheless, further improvement of the temporal resolution might result in more accurate (and possibly higher, as the measured value is an average flow measure over the entire duration of the measurement) flow reserve values in healthy persons, which will increase the ability to discriminate more patients from healthy controls. With our current equipment, however, parallel imaging acceleration factors above 2 results in a strong decrease in image quality.

Along the same vein, the temporal resolution of the waveform acquisition and the accuracy of the flow values can be improved, particularly in healthy controls, by applying faster image sampling. However, a present drawback is that spatial resolution will consequently decrease.

### Conclusion

This exploratory study demonstrates that quantitative 2D MR cine PCI flow waveform measurement in the popliteal artery is a simple and stable method to assess the functional severity of arterial stenoses in patients with intermittent claudication. A strong reduction in maximum hyperemic flow and absolute flow reserve was found in patients with intermittent claudication compared to healthy controls. Therefore, assessment of flow reserve might be a valuable addition to MR angiography to objectively determine the hemodynamic consequences and disease severity in PAD patients.

## Supporting Information

Figure S1 **Overview of the imaging protocol.** Three flow measurements were acquired at rest, before provoking reactive hyperemia to determine the average resting flow and reproducibility of resting flow. Reactive hyperemia was provoked by a cuff paradigm. After cuff deflation, 10 flow measurements were acquired during reactive hyperemia to determine the listed flow reserve measures. ^*^CE-MRA was performed in patients only. In healthy controls flow measurements started 10 minutes after the survey was completed. ^**^Nominal scan duration at a regular heart rate of 60 beats per minute.
(TIF)

Figure S2 **Coronal (A) and sagittal (B) reconstructions of CE-MRA of the upper leg of a PAD patient, showing the superficial femoral and popliteal artery.** The cine PCI plane (line segments) was angulated perpendicular to the popliteal artery.
(TIF)

Figure S3 **Overview of the different flow (reserve) measures.** Absolute and relative flow reserve are defined as the absolute difference and the ratio between maximum hyperemic and resting flow, respectively. TTP, time-to-peak; TTR, time-to-recover.
(TIF)

Figure S4 **Example of PCI modulus images (left corner panel A and B) and phase images (right corner panel A and B) and the correspondingly measured 2D velocity profiles of a PAD patient (panel A) and healthy control (panel B).** The brightest pixels of the modulus images represent the popliteal artery and is located within the white box on the phase images. 2D velocity profiles represent peak systolic velocity across the popliteal artery. Maximum peak velocity values were 24.3 cm/s and 92.6 cm/s respectively for the patient and healthy control, respectively.
(TIF)

Figure S5 **Flow waveforms in a PAD patient (panel A) and a healthy control (panel B) at rest and during maximum hyperemia.** Note the mono-phasic flow waveform in the patient, both at rest and during reactive hyperemia.
(TIF)

Figure S6 **Peak flow in the popliteal artery before and after provoking reactive hyperemia by a cuff paradigm in a PAD patient (panel A) and a healthy control (panel B).** The shaded bar represents the period of cuff compression to provoke reactive hyperemia. There is close agreement between the two MRI readers for both the patient and healthy control subject.
(TIF)

Figure S7 **Resting flow (panel A) and absolute flow reserve (panel B) in PAD patients.** The upper and lower limits in the graphics represent the mean value ± 2SD of resting peak flow (panel A) and absolute peak flow reserve (panel B) of healthy controls. The cross-table (right C) shows the number of patients with flow and flow reserve values within (normal values) or below (impaired values) the lower limits.
(TIF)

Table S1 **Flow measures and reproducibility in patients with intermittent claudication and healthy controls.** Caption: values are mean ± SD. CV, coefficient of variation; RC, repeatability coefficient; ICC, intra-class correlation coefficient.
(DOCX)

Table S2 **Peak velocity, peak area and mean flow measures and reproducibility in patients with intermittent claudication and healthy controls.** Caption: values are mean ± SD. CV, coefficient of variation; RC, repeatability coefficient; ICC, intra-class correlation coefficient.
(DOCX)

Appendix S1 **Peak velocity, peak area and mean flow measures and reproducibility.**
(DOCX)
