# Supplementary figures and images for: MRI of Arterial Flow Reserve in Patients with Intermittent Claudication: Feasibility and Initial Experience
Source: PLoS One. 2012 Mar 8;7(3):e31514. doi: 10.1371/journal.pone.0031514 (PMC3297594; doi:10.1371/journal.pone.0031514)

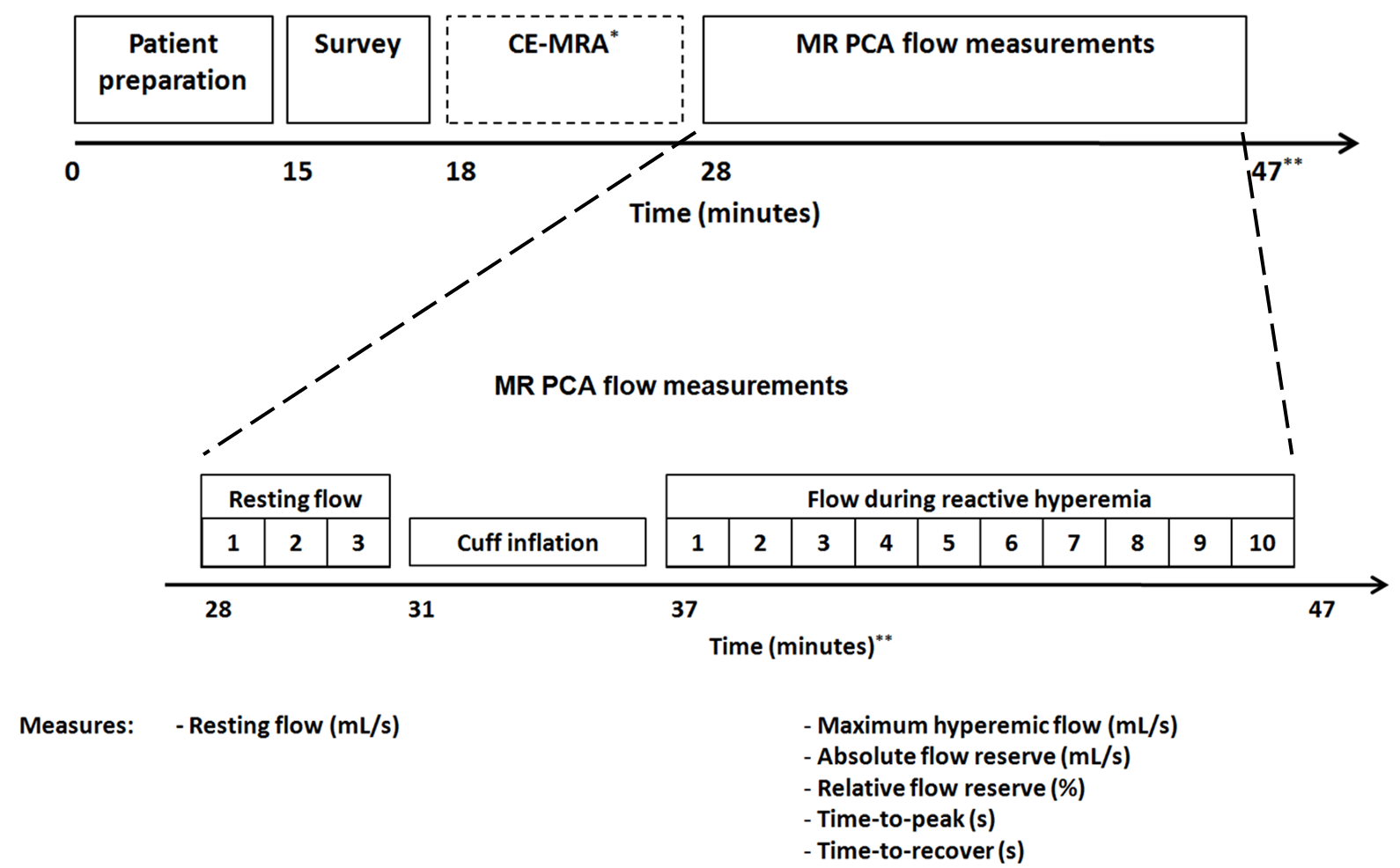

Supplement: Figure S1 — Overview of the imaging protocol. Three flow measurements were acquired at rest, before provoking reactive hyperemia to determine the average resting flow and reproducibility of resting flow. Reactive hyperemia was provoked by a cuff paradigm. After cuff deflation, 10 flow measurements were acquired during reactive hyperemia to determine the listed flow reserve measures. *CE-MRA was performed in patients only. In healthy controls flow measurements started 10 minutes after the survey was completed. **Nominal scan duration at a regular heart rate of 60 beats per minute. (TIF) [file pone.0031514.s001.tif]

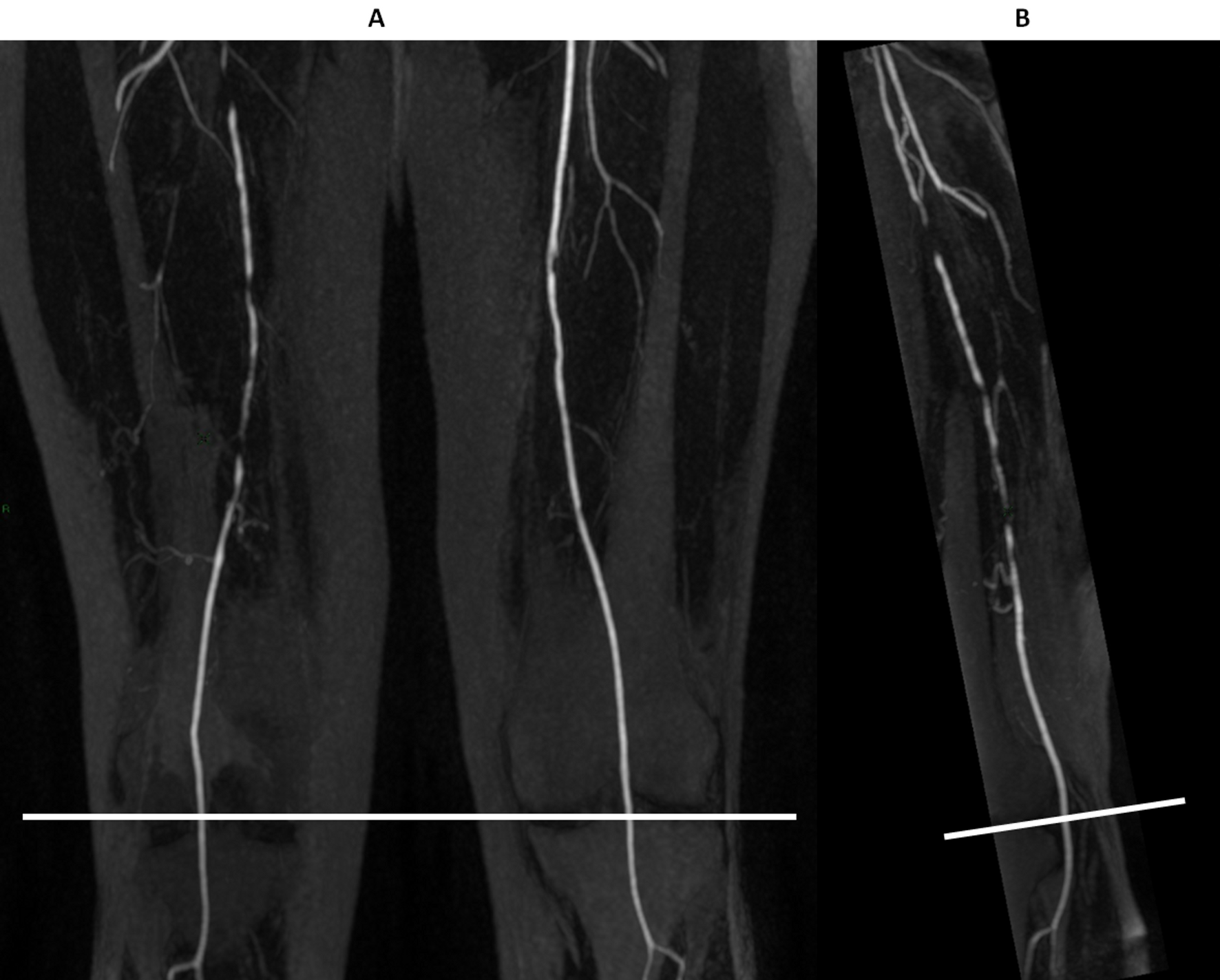

Supplement: Figure S2 — Coronal (A) and sagittal (B) reconstructions of CE-MRA of the upper leg of a PAD patient, showing the superficial femoral and popliteal artery. The cine PCI plane (line segments) was angulated perpendicular to the popliteal artery. (TIF) [file pone.0031514.s002.tif]

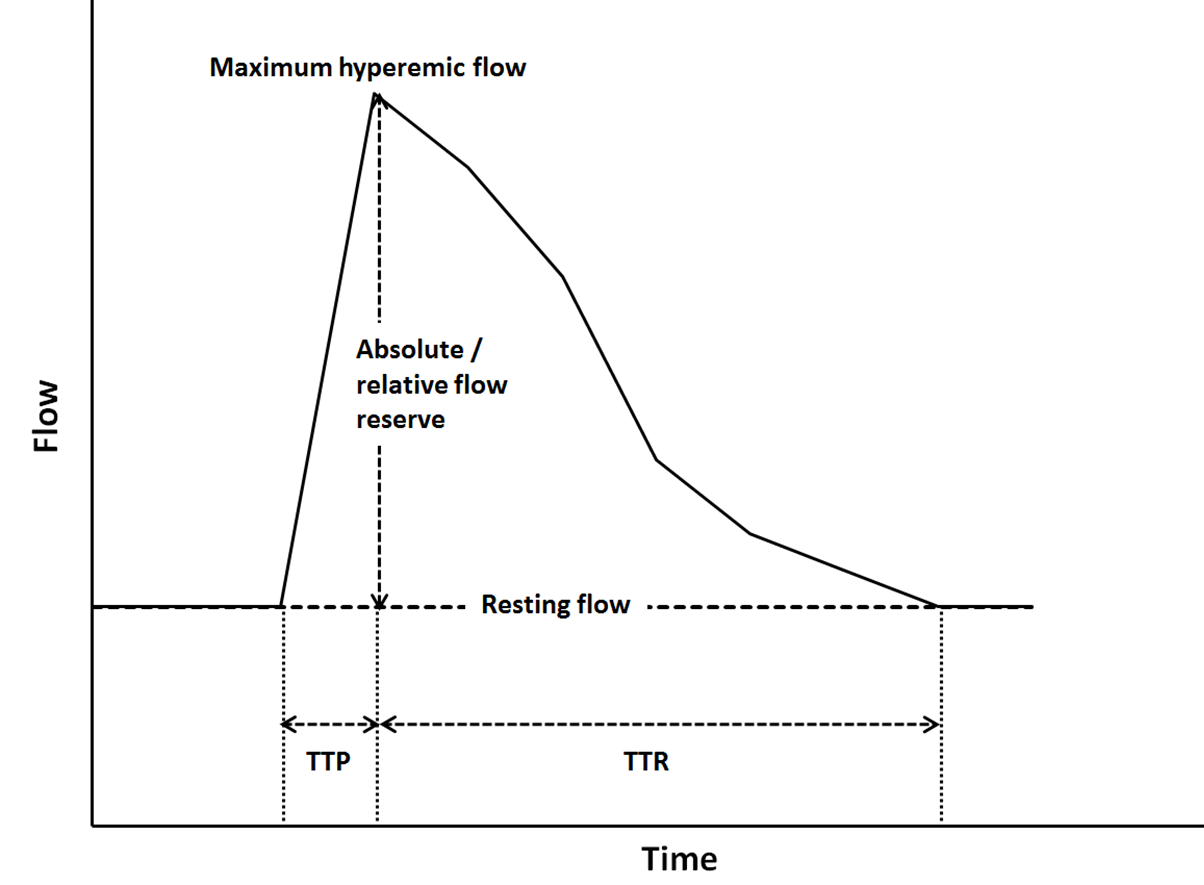

Supplement: Figure S3 — Overview of the different flow (reserve) measures. Absolute and relative flow reserve are defined as the absolute difference and the ratio between maximum hyperemic and resting flow, respectively. TTP, time-to-peak; TTR, time-to-recover. (TIF) [file pone.0031514.s003.tif]

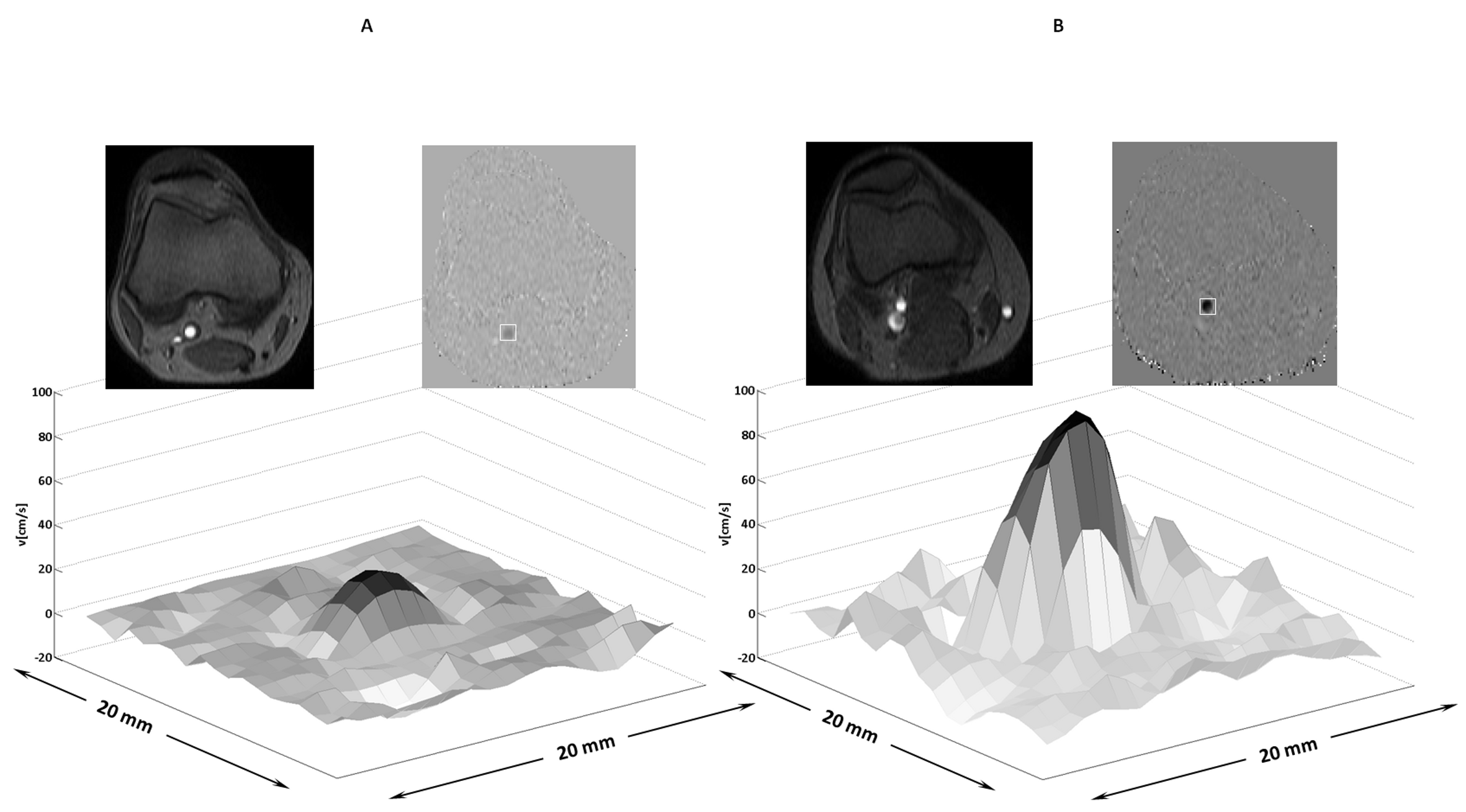

Supplement: Figure S4 — Example of PCI modulus images (left corner panel A and B) and phase images (right corner panel A and B) and the correspondingly measured 2D velocity profiles of a PAD patient (panel A) and healthy control (panel B). The brightest pixels of the modulus images represent the popliteal artery and is located within the white box on the phase images. 2D velocity profiles represent peak systolic velocity across the popliteal artery. Maximum peak velocity values were 24.3 cm/s and 92.6 cm/s respectively for the patient and healthy control, respectively. (TIF) [file pone.0031514.s004.tif]

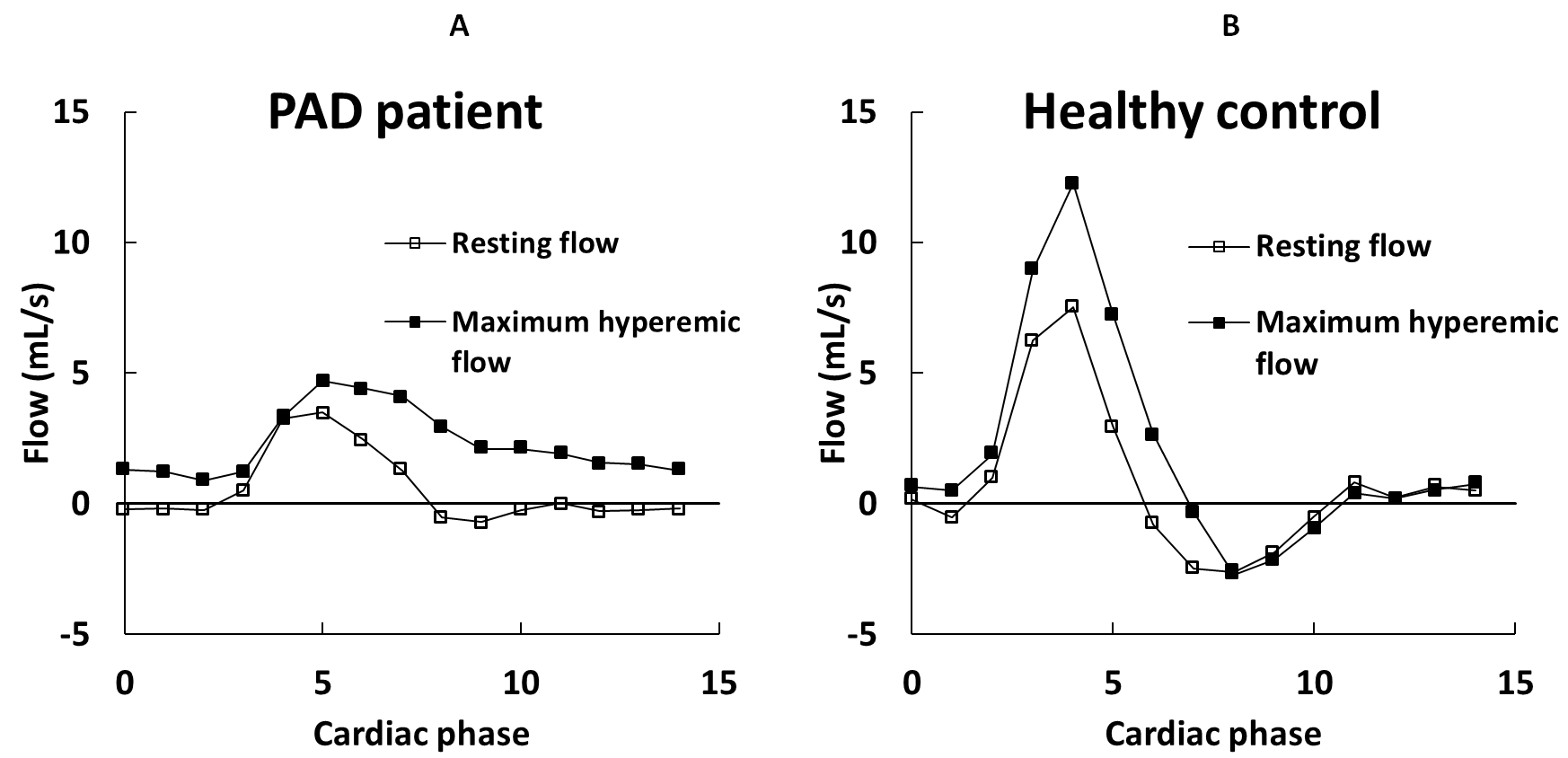

Supplement: Figure S5 — Flow waveforms in a PAD patient (panel A) and a healthy control (panel B) at rest and during maximum hyperemia. Note the mono-phasic flow waveform in the patient, both at rest and during reactive hyperemia. (TIF) [file pone.0031514.s005.tif]

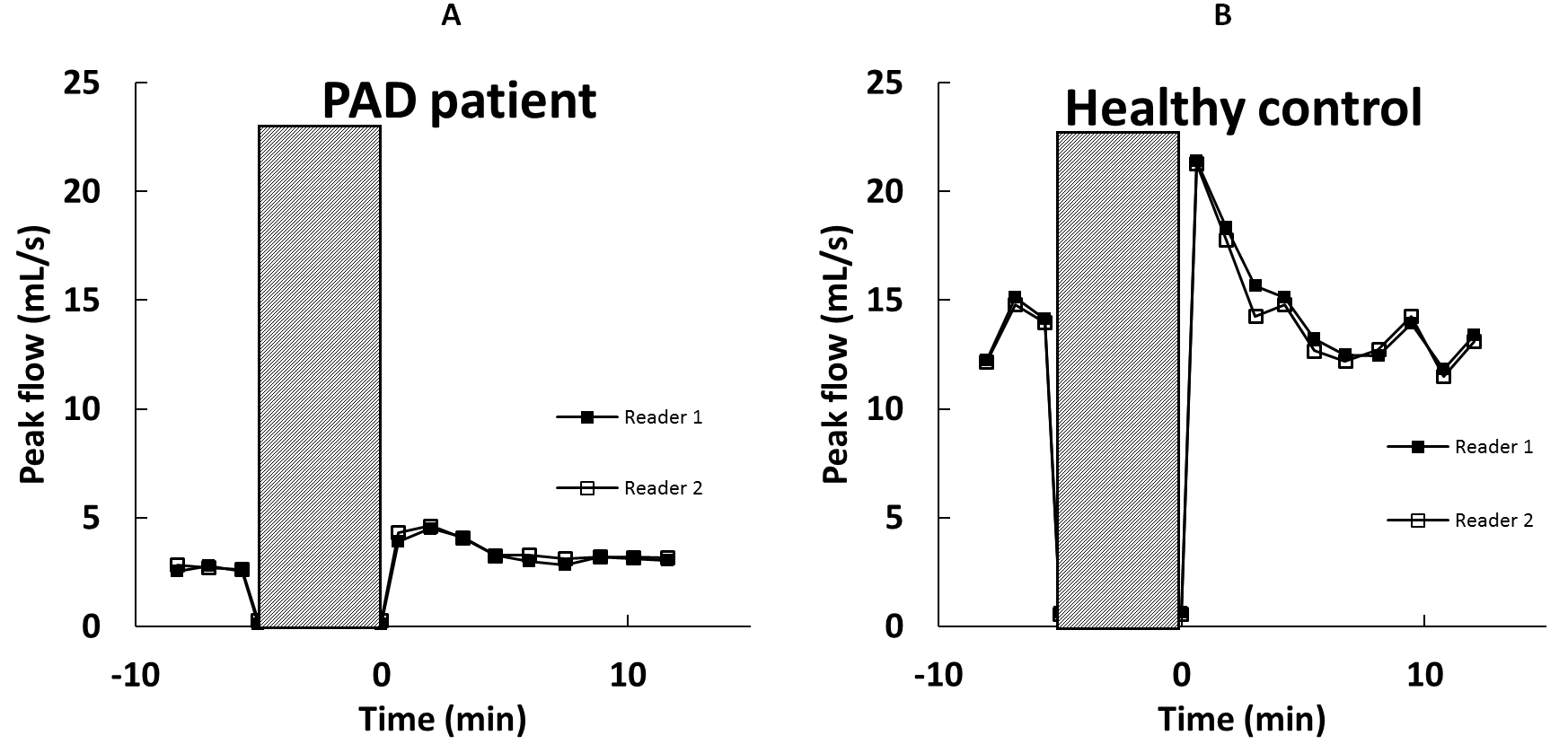

Supplement: Figure S6 — Peak flow in the popliteal artery before and after provoking reactive hyperemia by a cuff paradigm in a PAD patient (panel A) and a healthy control (panel B). The shaded bar represents the period of cuff compression to provoke reactive hyperemia. There is close agreement between the two MRI readers for both the patient and healthy control subject. (TIF) [file pone.0031514.s006.tif]

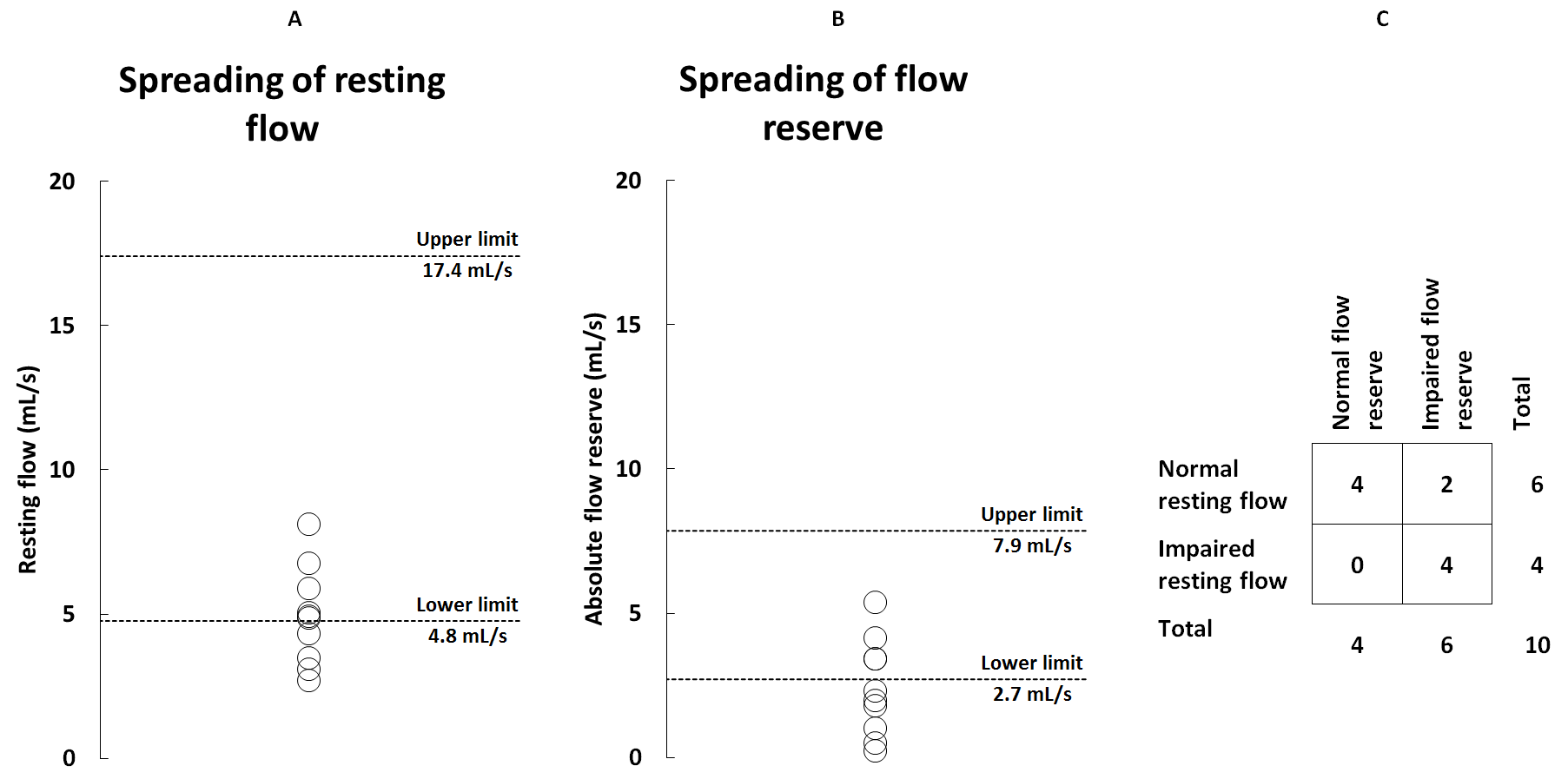

Supplement: Figure S7 — Resting flow (panel A) and absolute flow reserve (panel B) in PAD patients. The upper and lower limits in the graphics represent the mean value ± 2SD of resting peak flow (panel A) and absolute peak flow reserve (panel B) of healthy controls. The cross-table (right C) shows the number of patients with flow and flow reserve values within (normal values) or below (impaired values) the lower limits. (TIF) [file pone.0031514.s007.tif]
