# Supplementary material for: MRI of Arterial Flow Reserve in Patients with Intermittent Claudication: Feasibility and Initial Experience
Source: PLoS One. 2012 Mar 8;7(3):e31514. doi: 10.1371/journal.pone.0031514 (PMC3297594; doi:10.1371/journal.pone.0031514)
Supplement: Table S1 — Flow measures and reproducibility in patients with intermittent claudication and healthy controls. Caption: values are mean ± SD. CV, coefficient of variation; RC, repeatability coefficient; ICC, intra-class correlation coefficient. (DOCX) [file pone.0031514.s008.docx]

|  | | |  |  | **Resting flow** | |
| --- | --- | --- | --- | --- | --- | --- |
|  | | |  |  | **Patients** | **Healthy controls** |
|  | | |  |  | (n = 10) | (n = 10) |
| **Value** | | mL/s | | | 4.9 ± 1.6 | 11.1 ± 3.2 |
| **Interreader reproducibility** | | | | |  |  |
|  | **CV** | % | | | 4.0 | 4.3 |
|  | **RC** | mL/s | | | 0.5 | 1.0 |
|  | **ICC** | (95% CI) | | | 0.99 (0.96 - 0.99) | 0.99 (0.96 - 0.99) |
|  |  |  | | | **Maximum hyperemic flow** | |
|  | |  | | | **Patients** | **Healthy controls** |
| **Value** | | mL/s | | | 7.3 ± 2.9 | 16.4 ± 3.2 |
| **Interreader reproducibility** | | | | |  |  |
|  | **CV** | % | | | 4.6 | 3.5 |
|  | **RC** | mL/s | | | 0.9 | 1.6 |
|  | **ICC** | (95% CI) | | | 0.99 (0.95 - 0.99) | 0.97 (0.89 - 0.99) |
|  |  |  | | | **Absolute flow reserve** | |
|  | |  | | | **Patients** | **Healthy controls** |
| **Value** | | mL/s | | | 2.4 ± 1.6 | 5.3 ± 1.3 |
| **Interreader reproducibility** | | | | |  |  |
|  | **CV** | % | | | 9.8 | 8.7 |
|  | **RC** | mL/s | | | 0.6 | 1.2 |
|  | **ICC** | (95% CI) | | | 0.98 (0.93 - 0.99) | 0.89 (0.63 - 0.97) |
|  |  |  | | | **Relative flow reserve** | |
|  | |  | | | **Patients** | **Healthy controls** |
| **Value** | | % | | | 149 ± 31 | 151 ± 19 |
| **Interreader reproducibility** | | | | |  |  |
|  | **CV** | % | | | 3.1 | 3.4 |
|  | **RC** | % | | | 12.5 | 14.0 |
|  | **ICC** | (95% CI) | | | 0.98 (0.93 - 0.99) | 0.93 (0.76 - 0.98) |
