# Supplementary material for: MRI of Arterial Flow Reserve in Patients with Intermittent Claudication: Feasibility and Initial Experience
Source: PLoS One. 2012 Mar 8;7(3):e31514. doi: 10.1371/journal.pone.0031514 (PMC3297594; doi:10.1371/journal.pone.0031514)
Supplement: Table S2 — Peak velocity, peak area and mean flow measures and reproducibility in patients with intermittent claudication and healthy controls. Caption: values are mean ± SD. CV, coefficient of variation; RC, repeatability coefficient; ICC, intra-class correlation coefficient. (DOCX) [file pone.0031514.s009.docx]

|  | | |  |  | **Resting peak flow** | | **Resting peak velocity** | | **Resting peak area** | | **Resting mean flow** | |
| --- | --- | --- | --- | --- | --- | --- | --- | --- | --- | --- | --- | --- |
|  | | |  |  | **Patients** | **Healthy controls** | **Patients** | **Healthy controls** | **Patients** | **Healthy controls** | **Patients** | **Healthy controls** |
|  | | |  |  | (n = 10) | (n = 10) | (n = 10) | (n = 10) | (n = 10) | (n = 10) | (n = 10) | (n = 10) |
| **Value** | |  | | | 4.9 ± 1.6 (mL/s) | 11.1 ± 3.2 (mL/s) | 30.9 ± 14.9 (cm/s) | 53.7 ± 9.2 (cm/s) | 0.33 ± 0.12 (cm^2^) | 0.40 ± 0.07 (cm^2^) | 1.4 ± 0.5 (mL/s) | 1.3 ± 0.6 (mL/s) |
| **Interreader reproducibility** | | | | |  |  |  |  |  |  |  |  |
|  | **CV** | % | | | 4.0 | 4.3 | 0.6 | 2.3 | 15.1 | 7.1 | 6.4 | 5.3 |
|  | **RC** |  | | | 0.5 (mL/s) | 1.0 (mL/s) | 3.5 (cm/s) | 0.5 (cm/s) | 0.1 (cm^2^) | 0.1 (cm^2^) | 0.2 (mL/s) | 0.2 (mL/s) |
|  | **ICC** | (95% CI) | | | 0.99 (0.96 - 0.99) | 0.99 (0.96 - 0.99) | 0.98 (0.94 - 0.99) | 0.99 (0.99 - 0.99) | 0.86 (0.84 - 0.99) | 0.83 (0.45 - 0.95) | 0.99 (0.96 - 0.99) | 0.99 (0.95 - 0.99) |
|  |  |  | | | **Maximum hyperemic peak flow** | | **Maximum hyperemic peak velocity** | | **Maximum hyperemic peak area** | | **Maximum hyperemic mean flow** | |
|  | |  | | | **Patients** | **Healthy controls** | **Patients** | **Patients** | **Patients** | **Healthy controls** | **Patients** | **Healthy controls** |
| **Value** | |  | | | 7.3 ± 2.9 (mL/s) | 16.4 ± 3.2 (mL/s) | 40.9 ± 20.0 (cm/s) | 76.8 ± 14.4 (cm/s) | 0.35 ± 0.10 (cm^2^) | 0.41 ± 0.07 (cm^2^) | 3.9 ± 1.6 (mL/s) | 3.0 ± 1.4 (mL/s) |
| **Interreader reproducibility** | | | | |  |  |  |  |  |  |  |  |
|  | **CV** | % | | | 4.6 | 3.5 | 0.7 | 0.0 | 13.3 | 9.7 | 17 | 7.9 |
|  | **RC** |  | | | 0.9 (mL/s) | 1.6 (mL/s) | 0.8 (cm/s) | 0.0 (cm/s) | 0.1 (cm^2^) | 0.1 (cm^2^) | 1.9 (mL/s) | 0.7 (mL/s) |
|  | **ICC** | (95% CI) | | | 0.99 (0.95 - 0.99) | 0.97 (0.89 - 0.99) | 0.99 (0.99 - 0.99) | 0.99 (0.99 - 0.99) | 0.95 (0.81 - 0.99) | 0.76 (0.29 - 0.93) | 0.90 (0.65 - 0.97) | 0.99 (0.95 - 0.99) |
|  |  |  | | | **Absolute peak flow reserve** | | **Absolute peak velocity reserve** | | **Absolute peak area reserve** | | **Absolute mean flow reserve** | |
|  | |  | | | **Patients** | **Healthy controls** | **Patients** | **Patients** | **Patients** | **Healthy controls** | **Patients** | **Healthy controls** |
| **Value** | |  | | | 2.4 ± 1.6 (mL/s) | 5.3 ± 1.3 (mL/s) | 10.0 ± 7.7 (cm/s) | 23.1 ± 12.7 (cm/s) | 0.02 ± 0.04 (cm^2^) | 0.01 ± 0.04 (cm^2^) | 2.6 ± 1.6 (mL/s) | 1.7 ± 1.1 (mL/s) |
| **Interreader reproducibility** | | | | |  |  |  |  |  |  |  |  |
|  | **CV** | % | | | 9.8 | 8.7 | 3.9 | 5.3 | 102 | 259 | 23.3 | 15.7 |
|  | **RC** |  | | | 0.6 (mL/s) | 1.2 (mL/s) | 1.1 (cm/s) | 3.4 (cm/s) | 0.1 (cm^2^) | 0.1 (cm^2^) | 1.7 (mL/s) | 0.7 (mL/s) |
|  | **ICC** | (95% CI) | | | 0.98 (0.93 - 0.99) | 0.89 (0.63 - 0.97) | 0.99 (0.99 - 0.99) | 0.99 (0.97 - 0.99) | 0.56 (0.00 - 0.87) | 0.53 (0.00 - 0.86) | 0.90 (0.66 - 0.98) | 0.96 (0.89 - 0.99) |
|  |  |  | | | **Relative peak flow reserve** | | **Relative peak velocity reserve** | | **Relative peak area reserve** | | **Relative mean flow reserve** | |
|  | |  | | | **Patients** | **Healthy controls** | **Patients** | **Patients** | **Patients** | **Healthy controls** | **Patients** | **Healthy controls** |
| **Value** | | % | | | 149 ± 31 | 151 ± 19 | 133 ± 21 | 145 ± 26 | 109 ± 13 | 103 ± 10 | 320 ± 147 | 243 ± 86 |
| **Interreader reproducibility** | | | | |  |  |  |  |  |  |  |  |
|  | **CV** | % | | | 3.1 | 3.4 | 1.3 | 2.4 | 8.4 | 8.5 | 8.2 | 19.4 |
|  | **RC** | % | | | 12.5 | 14.0 | 4.9 | 9.8 | 25 | 24 | 72 | 130 |
|  | **ICC** | (95% CI) | | | 0.98 (0.93 - 0.99) | 0.93 (0.76 - 0.98) | 0.99 (0.99 - 0.99) | 0.98 (0.93 - 0.99) | 0.48 (0.00 - 0.84) | 0.49 (0.00 - 0.84) | 0.98 (0.91 - 0.99) | 0.77 (0.31 - 0.94) |
