# Supplementary material for: MRI of Arterial Flow Reserve in Patients with Intermittent Claudication: Feasibility and Initial Experience
Source: PLoS One. 2012 Mar 8;7(3):e31514. doi: 10.1371/journal.pone.0031514 (PMC3297594; doi:10.1371/journal.pone.0031514)
Supplement: Appendix S1 — Peak velocity, peak area and mean flow measures and reproducibility. (DOCX) [file pone.0031514.s010.docx]

**Appendix S1**

In addition to arterial peak flow also mean flow, peak velocity and area were determined and are listed in table S2.

*Mean flow*. No significant differences for mean flow were found between patients and healthy controls (table S2). The results in table S2 also confirm that interreader reproducibility is markedly lower for mean flow as compared to peak flow. Previous studies already revealed better discriminative capabilities and reproducibility for arterial peak flow over mean flow, which is confirmed in the current study [[2](#_ENREF_2),[29](#_ENREF_29)]. Poor interreader reproducibility for mean flow is probably induced by low contrast between the lumen of the popliteal artery during diastole and static tissue, which makes it difficult to correctly identify vessel contours. Also, the applied acquisition settings, especially the relatively high phase encoding velocity (VENC) of 100 cm/s is less suitable to accurately measure low flow velocities during diastole in the popliteal artery. Nevertheless, we choose this VENC value as we were primarily focused on peak flow. A venc value of 100 cm/s was approx. 30-40% above the highest peak velocity values we found at rest in healthy controls in a previous study [[29](#_ENREF_29)], and we expected this value to be high enough to measure the maximum hyperemic peak velocity. We decided to use a fixed (i.e. predefined and constant) phase encoding velocity for both patients and controls as we (i) we did not know the optimal phase encoding velocity for each patient (especially during hyperemia) prior to the study, and (ii) in clinical practice, a fixed phase encoding velocity would be highly desirable for reasons of time efficiency, and also (iii) by using a constant phase encoding velocity the methodology was identical for each subject.

*Peak velocity*. Peak velocity results are concordant to peak flow results, with resting peak velocity, maximum hyperemic peak velocity and absolute peak velocity reserve being significantly lower in patients (p < 0.01), whereas no significant difference was found for relative peak velocity reserve. Interreader reproducibility is excellent, which is hardly surprising, as during data analysis it is easy to identify the intraluminal voxel with the highest velocity (i.e at the vessel’s center). Nevertheless, in clinical practice the use of peak velocity might be problematic. Arterial peak velocity is known to increase over hemodynamically significant stenotic vessel segments (an increase that is measured clinically by determining the peak systolic velocity (PSV)-ratio by ultrasound [[48](#_ENREF_48)]). As far as peak flow is concerned, the increase in arterial peak flow is largely neutralized by the decrease in area at the stenosis. Moreover, during hyperemia, the arterial peak velocity only measures the increase in velocity, but not the influence of vasodilation. By measuring arterial peak flow, both the increase in velocity and vasodilatation are considered, which makes peak flow a more robust and physiologically more relevant measure. In addition peak velocity is much more sensitive to errors in angulation between imaging plane and vessel axis. Small errors in non-perpendicular orientation between vessel and imaging plane will have no impact upon flow as cross-sectional area increases compensate the decreases in the axial velocity component.

*Cross-sectional area.* Vessel cross-sectional area showed no significant differences between patients and healthy controls and is therefore not useful to discriminate patients with PAD from healthy controls.

References:

48. de Smet AA, Ermers EJ, Kitslaar PJ (1996) Duplex velocity characteristics of aortoiliac stenoses. J Vasc Surg 23: 628-636.
